# Pressure Overload in Mice With Haploinsufficiency of Striated Preferentially Expressed Gene Leads to Decompensated Heart Failure

**DOI:** 10.3389/fphys.2018.00863

**Published:** 2018-07-10

**Authors:** Chang Shu, He Huang, Ying Xu, Marcello Rota, Andrea Sorrentino, Yuan Peng, Robert F. Padera, Virginia Huntoon, Pankaj B. Agrawal, Xiaoli Liu, Mark A. Perrella

**Affiliations:** ^1^Division of Pulmonary and Critical Care Medicine, Department of Medicine, Brigham and Women’s Hospital, Harvard Medical School, Boston, MA, United States; ^2^Respiratory Center, Children’s Hospital, Chongqing Medical University, Chongqing, China; ^3^Department of Anesthesiology, The Second Affiliated Hospital, Chongqing Medical University, Chongqing, China; ^4^Department of Anesthesiology, Children’s Hospital, Chongqing Medical University, Chongqing, China; ^5^Department of Anesthesia, Brigham and Women’s Hospital, Harvard Medical School, Boston, MA, United States; ^6^Department of Physiology, New York Medical College, Valhalla, NY, United States; ^7^Cardiovascular Medicine, Department of Medicine, Brigham and Women’s Hospital, Harvard Medical School, Boston, MA, United States; ^8^Division of Health Sciences and Technology, Harvard-MIT Health Sciences and Technology, Cambridge, MA, United States; ^9^Department of Pathology, Brigham and Women’s Hospital, Harvard Medical School, Boston, MA, United States; ^10^Divisions of Newborn Medicine and Genetics & Genomics, Manton Center for Orphan Disease Research, Boston Children’s Hospital, Harvard Medical School, Boston, MA, United States; ^11^Department of Pediatric Newborn Medicine, Brigham and Women’s Hospital, Harvard Medical School, Boston, MA, United States

**Keywords:** myosin light chain kinase, striated preferentially expressed gene, haploinsufficiency, pressure overload, decompensated heart failure, fibrosis

## Abstract

Striated preferentially expressed gene (Speg) is a member of the myosin light chain kinase family of proteins. Constitutive Speg deficient (Speg^−/−^) mice develop a dilated cardiomyopathy, and the majority of these mice die *in utero* or shortly after birth. In the present study we assessed the importance of Speg in adult mice. Speg^−/−^ mice that survived to adulthood, or adult striated muscle-specific Speg knockout mice (Speg-KO), demonstrated cardiac dysfunction and evidence of increased left ventricular (LV) internal diameter and heart to body weight ratio. To determine whether heterozygosity of Speg interferes with the response of the heart to pathophysiologic stress, Speg^+/−^ mice were exposed to pressure overload induced by transverse aortic constriction (TAC). At baseline, Speg^+/+^ and Speg^+/−^ hearts showed no difference in cardiac function. However, 4 weeks after TAC, Speg^+/−^ mice had a marked reduction in LV function. This defect was associated with an increase in LV internal diameter and enhanced heart weight to body weight ratio, compared with Speg^+/+^ mice after TAC. The response of Speg^+/−^ mice to pressure overload also included increased fibrotic deposition in the myocardium, disruption of transverse tubules, and attenuation in cell contractility, compared with Speg^+/+^ mice. Taken together, these data demonstrate that Speg is necessary for normal cardiac function and is involved in the complex adaptation of the heart in response to TAC. Haploinsufficiency of Speg results in decompensated heart failure when exposed to pressure overload.

## Introduction

The myosin light chain kinase (MLCK) family of proteins plays important roles in the structure and regulation of cytoskeletal function in myocytes (Sutter et al., 2004). The MLCK subfamily UNC-89, named after the kinase found in Caenorhabditis elegans, includes the vertebrate proteins striated preferentially expressed gene (Speg) and obscurin. Previous investigations have reported the importance of UNC-89 in sarcomere assembly of muscle cells, as loss of function of UNC-89 leads to disorganization of myosin thick filaments (Qadota et al., 2008; Wilson et al., 2012). Moreover, obscurin deficiency in mice results in an alteration in the sarcoplasmic reticulum (SR) of myocytes, and obscurin is the link between the sarcomere and the SR (Lange et al., 2009).

The Speg gene is part of a muscle-specific gene locus (Hsieh et al., 2000; Tam et al., 2006), with Spegα and Spegβ being specifically expressed in striated muscle (cardiac and skeletal). We have previously shown that a lack of Speg due to a mutation of the Speg gene (Speg^−/−^) on a C57BL/6 genetic background leads to a dilated cardiomyopathy and embryonic/perinatal mortality (Liu et al., 2009, 2015). However, a small percentage of Speg^−/−^ pups remains viable at 3 weeks of age in the settings of heterozygous breeding on a C57BL/6 × 129 SvJ genetic background (Liu et al., 2009, 2015). Electron microscopy of Speg^−/−^ hearts at 18.5 days post coitum revealed evidence of myofibril disarray with thin, loosely arranged, and disorganized myofibrils (Liu et al., 2009). Speg mutations have been found in patients with centronuclear myopathy (Agrawal et al., 2014), and skeletal muscle from Speg mutant mice revealed evidence for centrally placed nuclei. Interestingly, this condition was also found in skeletal muscles of obscurin knockout mice (Lange et al., 2009), supporting the notion that UNC-89 subfamily members are critical components of sarcomere assembly.

In the hearts of Speg^−/−^ mice, we found reduced myocyte maturation (Legato, 1970; Markwald, 1973; Ono et al., 2005) and density suggesting that myocardial homeostasis is defective in these animals (Liu et al., 2009, 2015). Further investigation in the compartment of cardiac progenitor cells (CPCs) revealed that lack of Speg interfered with clone formation, growth, and differentiation *in vitro* (Liu et al., 2015). Importantly, administration of wild-type CPCs into the hearts of Speg^−/−^ fetuses resulted in CPC engraftment and differentiation, myocardial maturation, and rescue of Speg^−/−^ mice from neonatal heart failure. These findings document that Speg is necessary for proper myocyte formation and maturation, and for cardiac development.

In spite of the recognized function of Speg in the developing heart (Liu et al., 2009, 2015), the role of Speg in adult life, under normal or pathological circumstances, is less clear. Only recently, it has been reported that acute loss of Speg leads to heart failure in adult mice and is associated with a disruption in transverse tubule integrity, calcium handling, and junctional membrane complex activity (Quick et al., 2017). These findings are consistent with the fact that Speg deficiency in the skeletal muscle compartment results in abnormal triad development (formed by transverse tubules and sarcoplasmic reticulum), and faulty calcium handling and excitation-contraction coupling (Huntoon et al., 2018). In the present study we assessed cardiac function in the small cohort of Speg^−/−^ mice that survived to adulthood (Liu et al., 2009) and in conditional Speg knockout (Speg-KO) mice (Huntoon et al., 2018). The striated-muscle specific disruption of the gene allowed us to circumvent problems related to embryonic and perinatal mortality. In addition, to assess the consequences of reduced Speg expression on the response of the heart to pressure overload, Speg^+/−^ mice exposed to transverse aortic constriction (TAC) were studied. We report that surviving adult Speg^−/−^ mice demonstrated cardiac dysfunction, and that conditional deletion of Speg in Speg-KO animals resulted in dilated cardiomyopathy. In addition, mice with haploinsufficiency of Speg developed an impaired compensatory response to pressure overload, with decompensated heart failure 4 weeks after TAC.

## Materials and Methods

### Speg Mutant Mice

Speg^−/−^ (mutant) mice were previously generated on a mixed 129SvJ and C57BL/6 genetic background as described (Liu et al., 2009). The initial assessment of cardiac function in mice that survived to adulthood (10–12 weeks of age) was performed on the offspring of breeding Speg^+/−^ mice (129SvJ x C57BL/6). The mice were subsequently backcrossed 9 consecutive generations to yield Speg^−/−^ mice on a pure C57BL/6 genetic background. On this genetic background, Speg^−/−^ mice die *in utero* or on the day of birth (Liu et al., 2009, 2015). Thus, studies assessing the effect of pressure overload on left ventricle (LV) function were only performed on Speg^+/+^ and Speg^+/−^ mice (C57BL/6).

To evaluate cardiac function, we also assessed striated muscle specific Speg-KO mice on a C57BL/6 genetic background (mean 8.8 weeks of age). Speg-KO mice were created using a cre-loxP strategy, targeting exons 14–17 of Speg (Huntoon et al., 2018). Floxed Speg mice were bred with mice expressing cre driven by the muscle creatinine kinase (MCK) promoter to generate Speg-KO mice. Expression of cre starts on embryonic day 17, reaches peak levels by postnatal day 10, and remains on for the remainder of the mouse life (Bruning et al., 1998). These mice allowed us to not have disruption of Speg during early development, and to assess adult cardiac function of Speg-KO mice on a pure C57BL/6 background (Huntoon et al., 2018).

### Transverse Aortic Constriction

Chronic pressure overload of the LV in mice was induced using a model of transverse aortic constriction (TAC) (Rockman et al., 1991; deAlmeida et al., 2010). In brief, 10-week old mice were anesthetized with a mixture of ketamine (100 mg/kg, IP) and xylazine (10 mg/kg IP). The neck and chest were shaved, and mice were placed in a supine position under body temperature control. A midline cervical incision was made to expose the trachea. Mice were intubated with an 18-guage tubing and ventilated with a tidal volume of 0.2 ml at a rate of 120 strokes/min using a rodent respirator (model #687, Harvard Apparatus Inc., Holliston, MA, United States). The first and second ribs were cut along the left sternum to expose transverse aorta, and constriction of the aortic arch, between the take off of the right and left carotid arteries, was performed by tying a ligature with 6-0 silk suture against a 25-gauge needle. The needle was promptly removed to produce a constriction of transverse aorta, and the chest was closed in layers. Buprenex (0.05–0.10 mg/kg, IP) was used for postoperative analgesia. The patency of the aorta was acutely confirmed by Doppler ultrasound (see section “Echocardiography”). For the sham operation, the aorta was not ligated. In a subgroup of mice, catheters were placed in the right and left carotid arteries, to assess the pressure gradient across the TAC (Rockman et al., 1991). The mice undergoing TAC and sham surgery were further studied after 4 weeks (14 weeks of age). The use of mice and the studies performed were carried out in accordance with the Public Health Service policy on the humane care and use of laboratory animals, and the protocol was approved by the Institutional Animal Care and Use Committee of Brigham and Women’s Hospital.

### Echocardiography

A Vevo 2100 high-resolution micro-ultrasound system and a 40-MHz probe (VisualSonics) were used for transthoracic echocardiography in anesthetized adult mice (isoflurane 1.0–1.5%, inhaled). The hearts were imaged in the two-dimensional parasternal short-axis view, and an M-mode echocardiogram of the mid-ventricle was recorded at the level of papillary muscles as previously described (Liu et al., 2009, 2015). Left ventricular fractional shortening (FS) % = (LVID,d – LVID,s)/LVID,d × 100, and left ventricular ejection fraction (EF) % = (LV vol, d – LV vol,s)/LV vol,d × 100 were measured. End-systolic and end-diastolic internal dimensions of the LV (LVID,s; LVID,d) were also assessed from the M-mode image.

### Cardiomyocyte Shortening Measurements

Isolated cardiomyocytes from the LV of Speg^+/+^ and Speg^+/−^ hearts after 4 weeks of TAC, or after sham surgery, were placed in a bath on the stage of CK2 (Olympus) microscope for evaluation of cell contractility. Cells were bathed continuously with Tyrode solution at 37°C containing (in mM) NaCl 140, KCl 5.4, MgCl2 1, HEPES 5, Glucose 5.5, and CaCl2 1.0 (pH 7.4, adjusted with NaOH). Measurements were collected in field-stimulated cells by video edge detection (VED-205, Crescent Electronics; PowerLab 8/35, Adinstruments). Contractions were elicited by rectangular depolarizing pulses (S88, Grass stimulators), 2 ms in duration and 1.5 times threshold in intensity, with platinum electrodes. Data were analyzed with LabChart software (Signore et al., 2015).

### Immunohistochemistry

#### Immunofluorescent Staining

Hearts from the mice were harvested, formalin fixed, dehydrated, and embedded in paraffin. Tissue sections, 3–5 μm in thickness, were cut and deposited on poly-lysine coated slides. To improve antigen recognition, the heart sections were microwaved in 10 mM Citrate Buffer (pH6.0) for 10 ∼ 12 min to retrieve antigens. Alternatively, for CD31 staining, sections were pressure cooked in Dako retrieval solution for 15 min. The primary antibodies targeting cardiac troponin I (Abcam, ab47003, 1:200), Speg [1:200 (6)], CD31 (Abcam, ab56299, 1:500), and smooth muscle α-actin (Sigma, A2547, 1:400) were then incubated with heart sections at 37°C for 1 h or at 4°C overnight, followed by Tetramethylrhodamine (TRITC, Life Technology 1: 500 ∼ 1000) conjugated secondary antibodies at 37°C for 1 h. Heart sections were also stained with Alex488-conjugated WGA (Life Technology, W11261, 1:200) as described (Crossman et al., 2015), to visualize cell borders and transverse tubule structures. Nuclei were stained with 4′,6-diamidino-2-phenylindole (DAPI, Sigma, D9542, 1:1000). Following the immunostaining, fluorescent microscopy imaging was performed.

#### Colorimetric Staining

Masson trichrome staining was performed on heart sections as described (Liu et al., 2006; Schissel et al., 2009). The sections were scanned with BZ-9000 (KEYENCE) and images were saved as TIFF format for subsequent analysis in Adobe Photoshop CS3 Extended 10.0. Focal areas of replacement fibrosis, and interstitial fibrosis, were assessed. Regions of focal fibrosis were calculated as the sum of discrete fibrotic areas/sum of LV cardiomyocyte areas. Interstitial fibrosis was calculated as the sum of fibrotic areas lining cardiomyocyte borders/sum of LV cardiomyocyte areas.

### Statistical Analysis

All data are shown as the mean ± SEM. For comparisons between two groups, we used Student’s unpaired *t*-test. For comparisons of more than two groups, one-way analysis of variance (ANOVA) was performed. Statistical significance for all comparisons was accepted at *P* < 0.05.

## Results

### Cardiac Dysfunction in Adult Speg Deficient Mice

**FIGURE 1:**
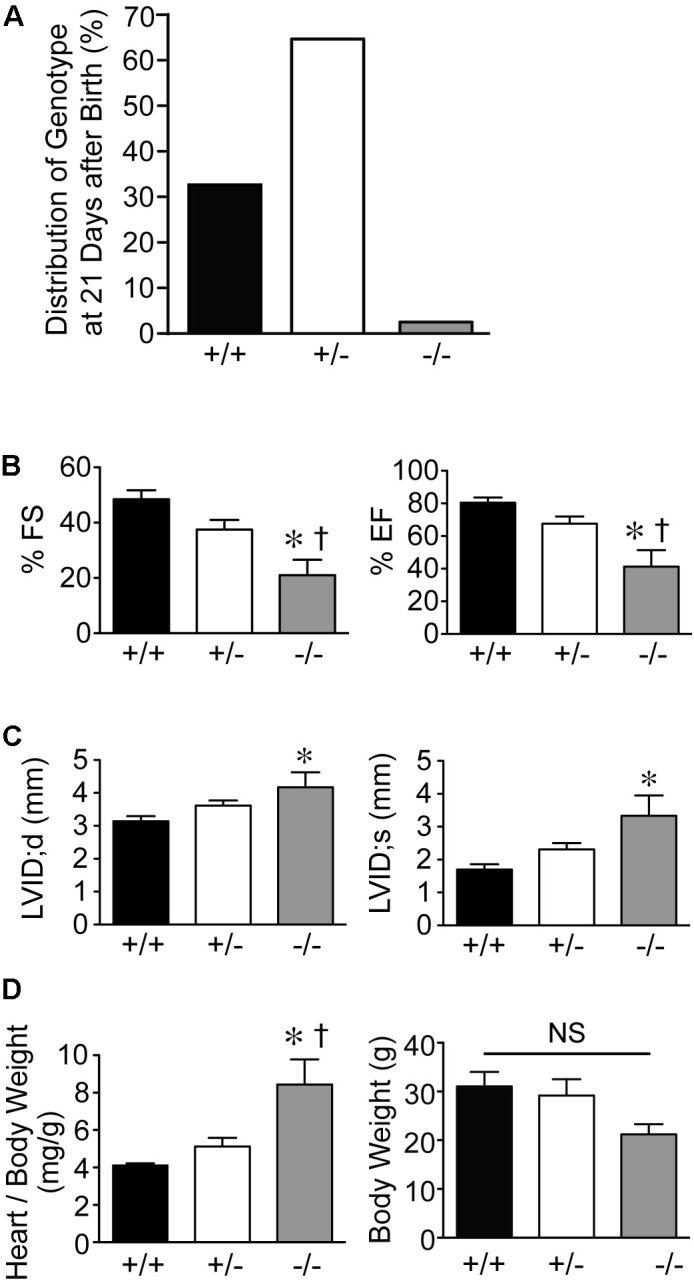
Dilated cardiomyopathy in adult Speg null mice. **(A)** Genotypes (%) of offspring from breeding of Speg heterologous mice on a C57BL/6 × 129Sv background. Speg wild type (+/+, black bar), heterologous (+/–, white bar), and null (–/–, gray bar) mice at the age of 21 days. Assessment of 305 total pups. **(B,C)** Echocardiography was performed on the surviving mice at 10–12 weeks of age (+/+, *n* = 5; +/–, *n* = 6; –/–, *n* = 6). **(B)** Left ventricular fractional shortening (FS, %) and ejection fraction (EF, %). **(C)** Left ventricular internal diameter (LVID, mm) in diastole and systole. Following echocardiography, mice were sacrificed, and heart and body weight were measured. **(D)** Ratio of heart to body weight (mg/g, left panel) and total body weight (g, right panel). Analysis performed by one-way ANOVA (*P* < 0.05), with significant comparisons ^∗^ versus +/+; ^†^ versus +/–. NS, not significant.

Genotypes of offspring from breeding of Speg^+/−^ mice on a C57BL/6 × 129Sv genetic background revealed only 2% of Speg^−/−^ are alive at 21 days of age (**Figure 1A**). Echocardiograms were performed on the surviving Speg^−/−^ mice. Speg^−/−^ mice at 10–12 weeks of age demonstrated a reduction in LV fractional shortening (FS) and LV ejection fraction (EF) compared with littermate Speg^+/+^ and Speg^+/−^ mice (**Figure 1B**). There was also evidence of ventricular dilatation in Speg^−/−^, with an increase in the LV internal diameter (LVID) in both diastole (d) and systole (s) (**Figure 1C**). At sacrifice, heart weight to body weight ratio was increased in Speg^−/−^ mice compared with Speg^+/+^ and Speg^+/−^ animals (**Figure 1D**). While the Speg^−/−^ mice show a tendency to be smaller, the body weights were not significantly different between the groups studied. Moreover, irrespective of body weight, the Speg^−/−^ hearts have a propensity to weigh more than Speg^+/+^ hearts (173 ± 21 mg versus 128 ± 11 mg, respectively, *P* = 0.05).

**FIGURE 2:**
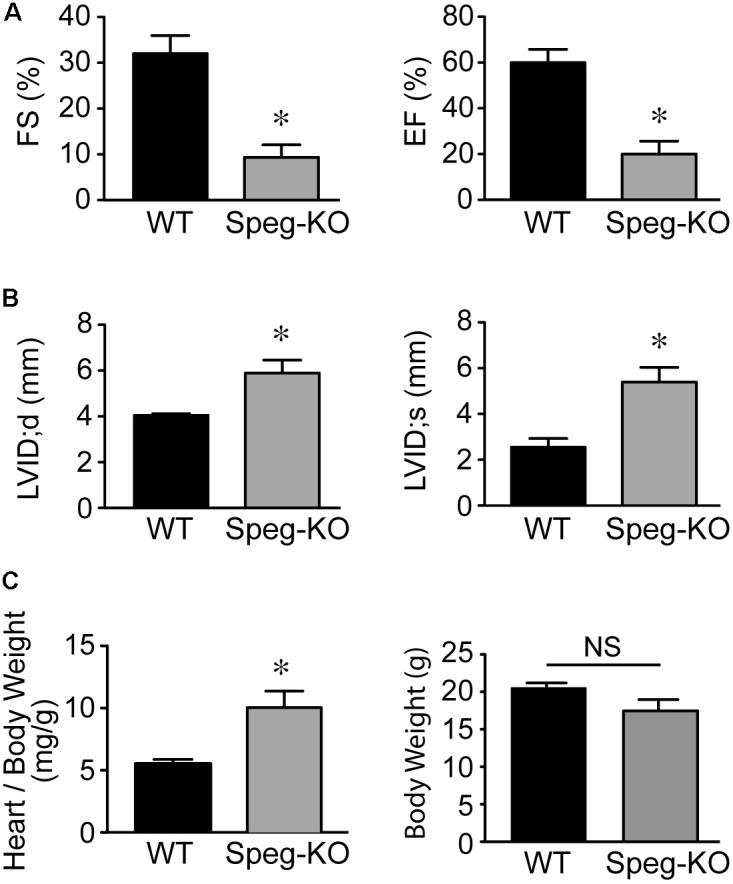
Dilated cardiomyopathy in adult Speg striated muscle-specific KO mice. Echocardiographic assessment of Speg wild type (WT, black bar, *n* = 4) and striated muscle specific null (KO, gray bar, *n* = 4) mice from 6 to 12 weeks of age. **(A)** Left ventricular fractional shortening (FS, %) and ejection fraction (EF, %). **(B)** Left ventricular internal diameter (LVID, mm) in diastole and systole. Following echocardiography, mice were sacrificed, and heart and body weight were measured. **(C)** Ratio of heart to body weight (mg/g, left panel) and total body weight (g, right panel). For **(A–C)**, analysis performed by Student’s unpaired *t*-test (*P* < 0.05), with significant comparison ^∗^ versus WT. NS, not significant.

Cardiac function of striated muscle specific Speg-KO mice (using MCK-cre) was evaluated. In these Speg deficient mice, echocardiography revealed a marked decrease in LVFS and LVEF compared with wild-type littermates (**Figure 2A**), whereas LVID during diastole and systole was increased (**Figure 2B**). Furthermore, heart weight to body weight ratio was also increased in Speg-KO mice (**Figure 2C**), whereas body weights were not different between the groups. In addition, not taking into account body weight, the Speg-KO hearts weighed more than the WT hearts (172 ± 22 mg vs. 114 ± 6 mg, respectively, *P* = 0.04). These data demonstrate a dilated cardiomyopathy in Speg deficient adult mice. Thus, loss of Speg results in ventricular dilation and reduced cardiac performance.

### Decompensation of LV Function in Adult Speg^+/−^ Mice Exposed to Pressure Overload

Because of the severity of the cardiac phenotype of mice lacking Speg, we employed Speg heterozygous animals to establish the role of Speg in the response of the heart to a stressful condition. The level of Speg transcripts in hearts of heterozygous mice was 19 ± 0.76%, with respect to wild-type mice, using quantitative real-time PCR. Adult Speg^+/−^ and Speg^+/+^ mice were exposed to pressure overload by TAC, or to sham surgery. After TAC, the pressure gradient across the banded aorta was analogous between Speg^+/−^ (61.6 mmHg) and Speg^+/+^ (61.3 mmHg) mice (Supplementary Figure S1). Echocardiography was then performed on Speg^+/−^ and Speg^+/+^ mice at 4 weeks after TAC or sham surgery.

**FIGURE 3:**
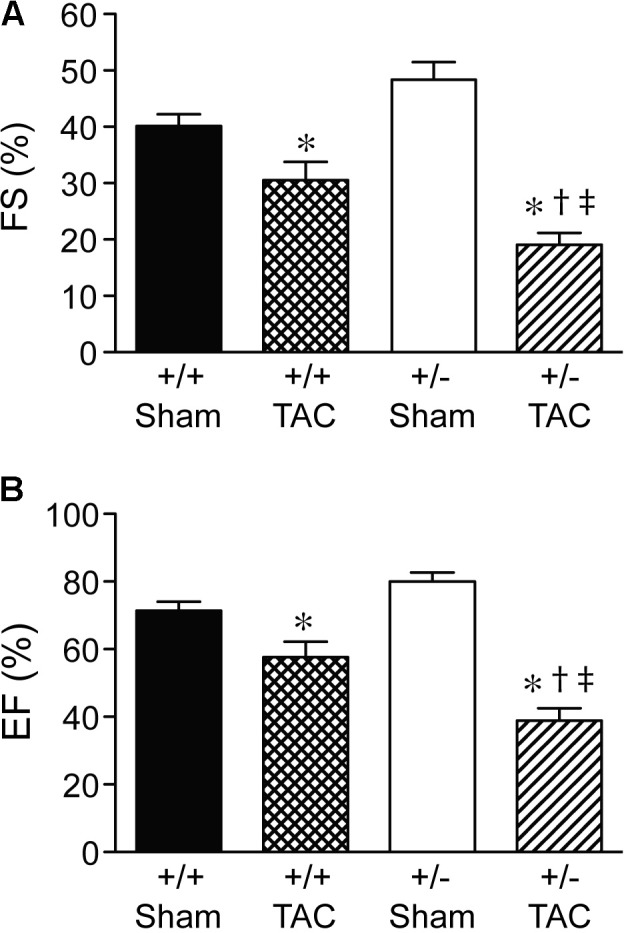
Decompensation of left ventricular function in Speg^+/−^ mice at 4 weeks post-TAC. Transverse aortic constriction (TAC) or sham surgery (sham) was performed on 8-week old Speg wild type (+/+) and heterozygous (+/–) mice. Echocardiography was performed on the mice (sham, *n* = 7/group; TAC, *n* = 7–11/group) after TAC or sham surgery. **(A)** Fractional shortening (FS, %) and **(B)** ejection fraction (EF, %) were assessed. Analysis by one-way ANOVA (*P* < 0.05), with significant comparisons ^∗^ versus sham of the same group; ^†^ versus Speg^+/+^ TAC; ^‡^ versus Speg^+/+^ sham.

**FIGURE 4:**
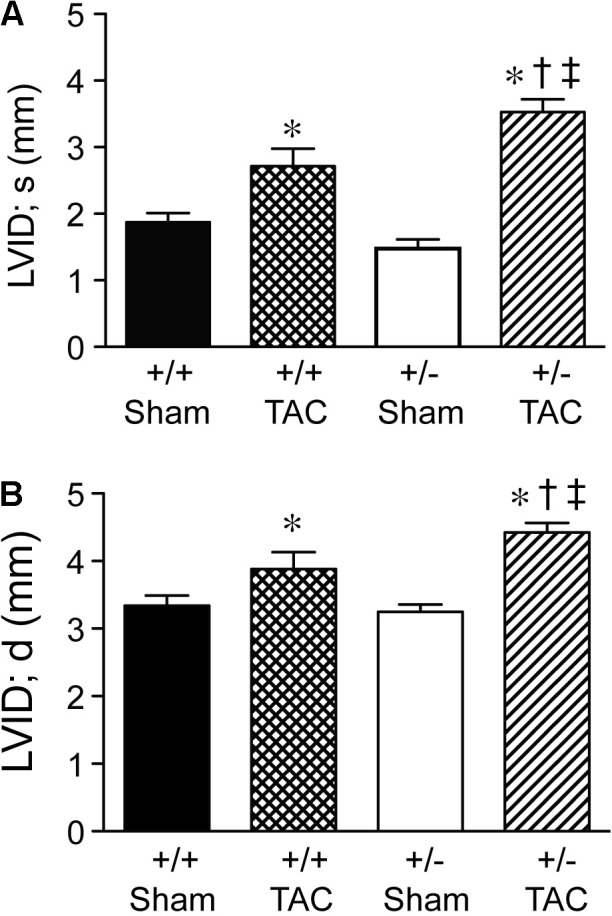
Left ventricular dilation at 4 weeks post-TAC in Speg^+/−^ mice. Transverse aortic constriction (TAC) or sham surgery (sham) was performed on 8-week old Speg wild type (+/+) and heterozygous (+/–) mice. Echocardiography was performed on the mice (sham, *n* = 7/group; TAC, *n* = 7–11/group) after TAC or sham surgery. Left ventricular internal diameter (LVID, mm) was assessed in **(A)** systole (s) and **(B)** diastole (d). Analysis by one-way ANOVA (*P* < 0.05), with significant comparisons ^∗^ versus sham of the same group; ^†^ versus Speg^+/+^ TAC; ^‡^ versus Speg^+/+^ sham.

At 4 weeks after TAC, the Speg^+/−^ mice had a decompensation in cardiac function compared with Speg^+/+^ mice, with a more marked decrease in LVFS and LVEF (**Figures 3A,B**, respectively). The functional parameters in Speg^+/−^ mice after TAC were also dramatically decreased compared with both sham groups (**Figure 3**). This decrease in LVFS and LVEF was associated with an increase in LVID (both systole and diastole, **Figures 4A,B**, respectively), consistent with LV chamber dilatation in Speg^+/−^ mice compared with Speg^+/+^ mice after TAC.

**FIGURE 5:**
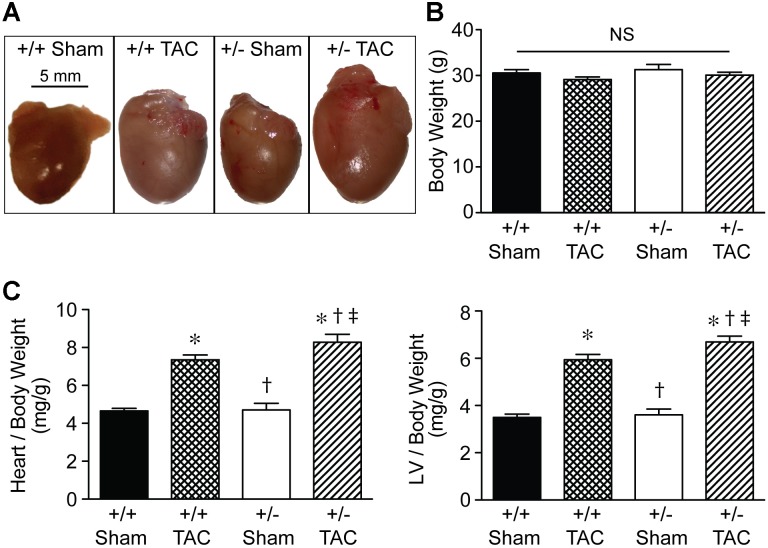
Heart size and cardiac hypertrophy of Speg^+/−^ mice post-TAC. Transverse aortic constriction (TAC) or sham surgery (sham) was performed on 10-week old Speg wild type (+/+) and heterozygous (+/–) mice. **(A)** Hearts were harvested 4 weeks after TAC, and representative macroscopic images of hearts from each group of mice were obtained. Scale bar represents 5 mm. **(B)** Total body weights (g) were assessed in each group. **(C)** Heart and left ventricle (LV) weights were measured, and ratios of heart or LV to body weight were analyzed at 4 weeks (sham, *n* = 7/group; TAC, *n* = 7–11/group) after TAC or sham surgery. Analysis by one-way ANOVA (*P* < 0.05), with significant comparisons ^∗^ versus sham of the same group; ^†^ versus Speg^+/+^ TAC; ^‡^ versus Speg^+/+^ sham.

Representative macroscopic images (**Figure 5A**) showed the increased size of Speg^+/−^ hearts compared with Speg^+/+^ hearts after TAC, and also compared with the hearts of Speg^+/−^ and Speg^+/+^ sham mice. Heart and LV weights in Speg^+/−^ hearts (247 ± 10 mg and 192 ± 8 mg, respectively) were greater than Speg^+/+^ hearts (214 ± 8 mg and 170 ± 5 mg, respectively, *P* < 0.05) after TAC. The overall body weights were not different between the groups (**Figure 5B**), and heart to body weight ratio, and LV to body weight ratio, demonstrated a significant increase in Speg^+/−^ hearts compared with Speg^+/+^ hearts after TAC and with hearts from the sham groups (**Figure 5C**). Taken together, these data suggest that at 4 weeks after TAC, Speg^+/−^ mice experience an impaired compensatory response to pressure overload with a reduction in cardiac function and an increase in LV chamber size, along with increased cardiac hypertrophy by weight, compared with Speg^+/+^ mice after TAC.

### Disrupted Transverse Tubular Structure and Increased Fibrotic Deposition in the Hearts of Speg^+/−^ Mice After TAC

**FIGURE 6:**
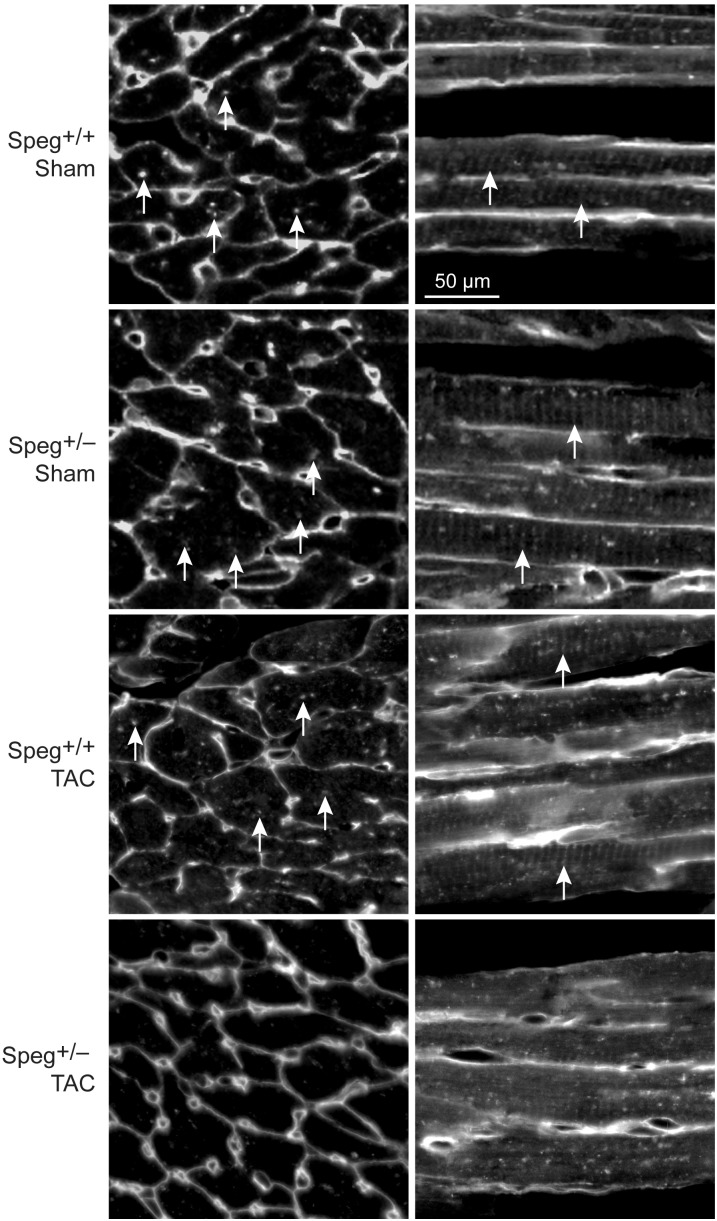
Altered transverse tubular structure in cardiomyocytes from the left ventricle of Speg^+/−^ mice post-TAC. Surface and transverse tubular membrane staining of sections from the left ventricle of Speg^+/+^ and Speg^+/−^ hearts, 4 weeks after sham or TAC surgery. Confocal images of cell membranes labeled with wheat germ agglutinin (WGA) are shown in transverse (left panels) and longitudinal (right panels) sections. Arrows point to transverse tubule structures. Scale bar, 50 μm.

**FIGURE 7:**
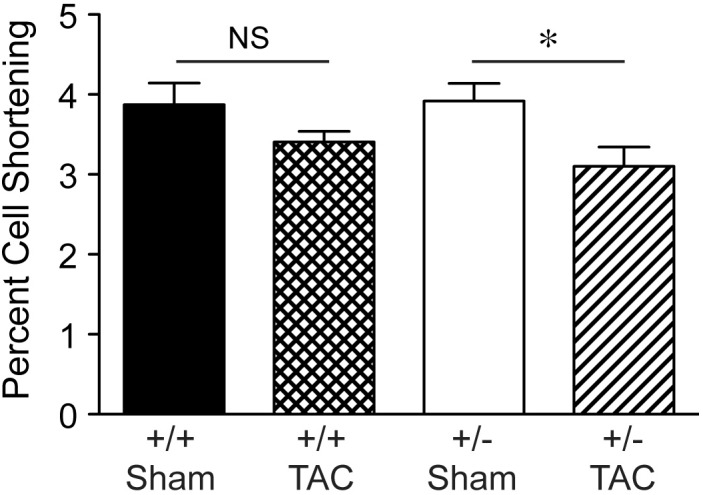
Decrease in percent cell shortening of Speg^+/−^ cardiomyocytes after TAC. Quantitative data for cell shortening of isolated cardiomyocytes from the LV of Speg^+/+^ and Speg^+/−^ mice exposed to TAC or sham for 4 weeks (Speg^+/+^ sham *n* = 124 from 4 mice; Speg^+/+^ TAC *n* = 199 from 5 mice; Speg^+/−^ sham *n* = 140 from 5 mice; Speg^+/−^ TAC *n* = 83 from 3 mice). Data are shown as mean ± SEM. Analysis by one-way ANOVA using Kruskal–Wallis test (*P* < 0.05), with significant comparison ^∗^ Speg^+/−^ TAC versus Speg^+/−^ sham. NS, not significant.

By histological assessment, the structure of cardiomyocytes, visualized by striations of cTnI staining, was similar in Speg^+/−^ and Speg^+/+^ hearts under normal physiologic conditions (Supplementary Figure S2). However, based on a previous study reporting that Speg deficiency in myocytes alters the structure of the transverse tubular system (Quick et al., 2017), we performed membrane labeling to assess the organization of the transverse tubules in myocytes at baseline or after exposure to pressure overload. The transverse tubular structure appeared intact in cardiomyocytes of both Speg^+/−^ and Speg^+/+^ sham hearts. However, under the pathophysiologic stress of pressure overload, the transverse tubular structure of Speg^+/−^ cardiomyocytes appeared less pronounced, with disruption of the tubular network compared with the distinct transverse tubules of Speg^+/+^ cardiomyocytes after 4 weeks of TAC (**Figure 6**, cross section and longitudinal views, white arrows). Because of the critical role of the t-tubular system in excitation contraction coupling in myocytes, we evaluated whether structural defects observed in Speg^+/−^ mice after TAC had functional implications. For this purpose, hearts from Speg^+/+^ and Speg^+/−^ mice were digested into single cell suspensions, and cell shortening was assessed. With respect to cells from sham-operated mice, percent of cell shortening was significantly decreased in cardiomyocytes from Speg^+/−^ hearts after TAC (**Figure 7**), an effect that was attenuated in cells from Speg^+/+^ mice.

**FIGURE 8:**
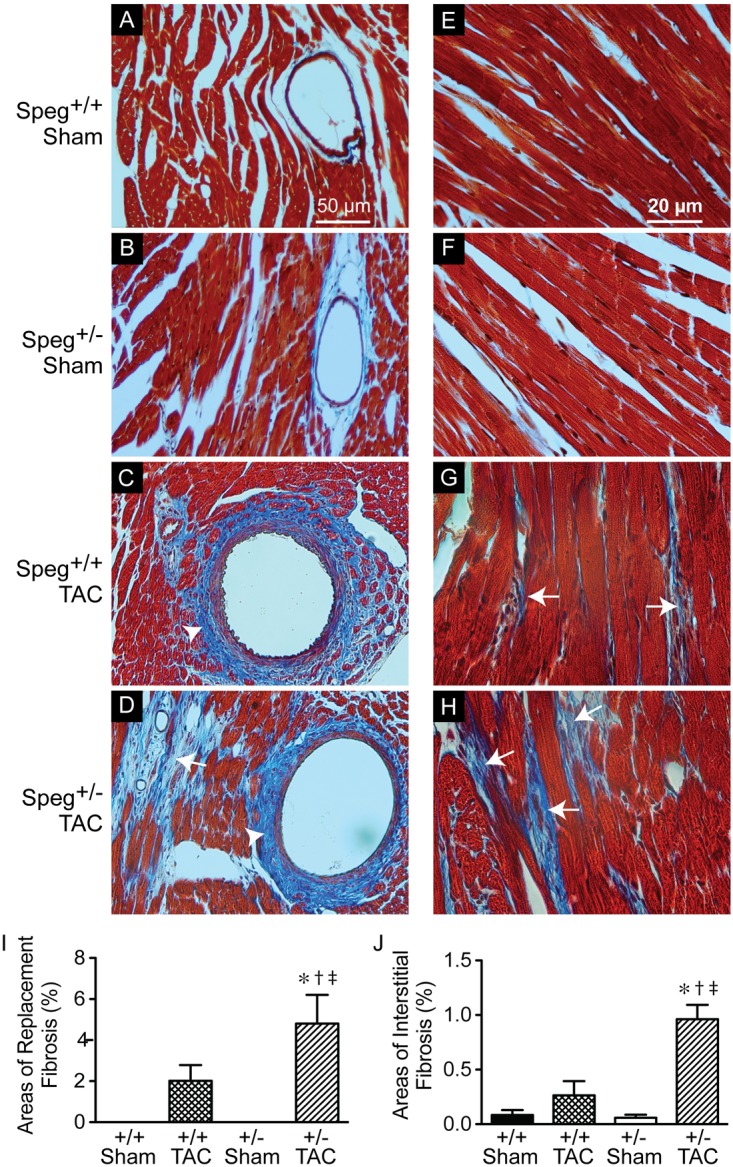
Increased areas of focal replacement fibrosis, and interstitial fibrosis, in the hearts of Speg^+/−^ mice post-TAC. Transverse aortic constriction (TAC) or sham surgery (sham) was performed on 8-week old Speg wild type (+/+) and heterozygous (+/–) mice. Hearts were harvested 4 weeks after TAC and histological analyses were performed. Representative images of hearts from Speg^+/+^ **(A,C,E,G)** and Speg^+/−^ **(B,D,F,H)** mice were stained by Masson’s Trichrome, in which collagen stains blue while the myocytes stain red. Scale bars, 50 μm **(A–D)** and 20 μm **(E–H)**. White arrows denote areas of focal replacement fibrosis **(D)** and interstitial fibrosis **(G,H)**. White arrowheads denote perivascular fibrosis **(C,D)**. Quantitation for percent area of focal replacement fibrosis **(I)** and interstitial fibrosis **(J)** from sections of the same hearts (*n* = 4 hearts/group) are shown in the bar graphs. Analysis by one-way ANOVA (*P* < 0.05), with significant comparisons ^∗^ versus sham of the same group; ^†^ versus Speg^+/+^ TAC; ^‡^ versus Speg^+/+^ sham.

**FIGURE 9:**
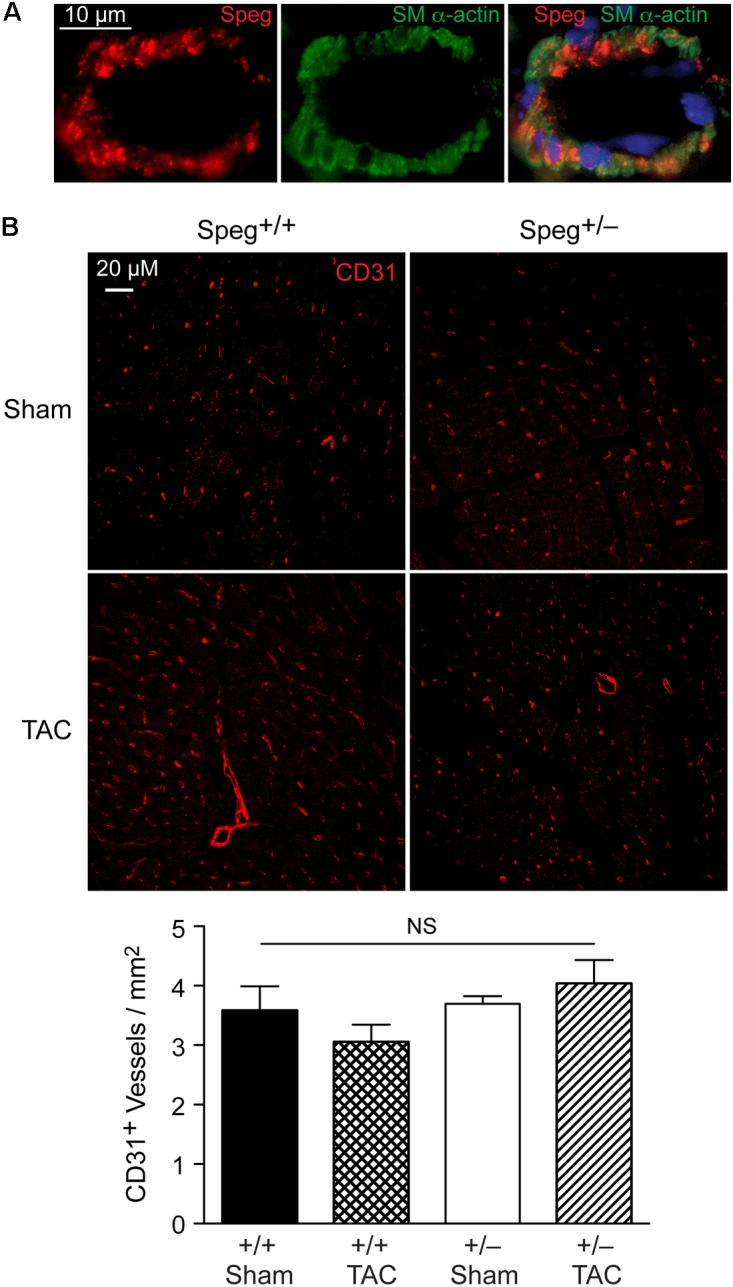
Speg is present in cardiac vessels of adult mouse hearts, and there is no difference in the number of vessels between Speg^+/+^ and Speg^+/−^ hearts at baseline or after TAC. **(A)** Representative image of fluorescent microscopy for Speg (red, left panel) and smooth muscle alpha-actin (SM α-actin, green, middle panel) staining of vascular smooth muscle cells from a vessel of an adult mouse heart. The vessels were also co-stained with 4′,6-diamidino-2-phenylindole (DAPI, blue) to identify nuclei (merged image, right panel). Scale bar, 10 μm. **(B)** Representative images of fluorescent microscopy for CD31 (red) staining of vessels from the left ventricles of Speg^+/+^ and Speg^+/−^ hearts, 4 weeks after sham or TAC surgery (upper panels). The lower panel (bar graph) shows quantification of CD31^+^ vessels per mm^2^ of tissue (sham, *n* = 4/group; TAC, *n* = 5/group). Analysis by one-way ANOVA (*P* = 0.277). NS, not significant.

To establish whether Speg haploinsufficiency was associated with alterations in myocardial composition, levels of fibrotic tissue were assessed in the various groups. Masson’s Trichrome staining performed on hearts after 4 weeks of TAC revealed increased areas of discrete replacement fibrosis (**Figure 8D**, white arrow) and interstitial fibrotic deposition (**Figures 8G,H**, white arrows) in Speg^+/−^ compared with Speg^+/+^ hearts. Sham hearts of both Speg^+/+^ and Speg^+/−^ mice revealed minimal fibrosis (**Figures 8A,B,E,F**). Quantitation of the areas of focal and interstitial fibrosis demonstrated a significant increase in Speg^+/−^ hearts after TAC compared with Speg^+/+^ hearts after TAC, and compared with sham hearts (**Figures 8I,J**). Assessment of the vasculature revealed evidence of perivascular fibrosis predominantly in large vessels in both Speg^+/−^ and Speg^+/+^ mice after TAC (**Figures 8C,D**, white arrowheads). While Speg is expressed in vessels of adult hearts (**Figure 9A**), staining for CD31 revealed that there was no difference in vessel numbers per tissue area between the groups (**Figure 9B**), and vessels appeared patent.

## Discussion

We have shown previously that mutation of the Speg gene locus in mice leads to cardiac dysfunction in newborn pups, and perinatal lethality (Liu et al., 2009). The dilated cardiomyopathy that developed in Speg^−/−^ pups was associated with a decrease in phosphorylated isoforms of tropomyosin, which together with actin play an important role in striated muscle contraction and relaxation. Quick et al. (2017) also recently showed that downregulation of Speg over 8 weeks, in cardiomyocyte specific tamoxifen-inducible Speg knockout mice, led to disruption of transverse tubular structure, impaired Ca^2+^ handling, altered excitation-contraction coupling, and heart failure. In the present study, we further demonstrate that Speg is critical for cardiac homeostasis in adult mice, as Speg deficient mice (either genetically absent throughout development with survival to adulthood, or striated muscle-specific loss of Speg starting at the time of birth) showed evidence of cardiac dysfunction (**Figures 1**, **2**). Moreover, mice haploinsufficient for Speg were susceptible to pressure overload after 4 weeks leading to decompensated heart failure (**Figures 3–5**). This phenotype in Speg^+/−^ mice undergoing TAC was also associated with an abnormality in transverse tubular structure (**Figure 6**), a decrease in cardiomyocyte contractility (**Figure 7**), and an increase in focal areas of replacement fibrosis and interstitial fibrosis (**Figure 8**).

The Speg locus contains four different isoforms (Hsieh et al., 2000). Spegα and Spegβ are expressed in striated muscle (cardiac and skeletal), whereas aortic preferentially expressed gene (Apeg)-1 and brain preferentially expressed gene (Bpeg) are expressed in smooth muscle (mainly vascular), in the aorta and brain, respectively (Hsieh et al., 1996, 1999, 2000; Sutter et al., 2004). In the original Speg mutant mouse (Liu et al., 2009), the gene targeting strategy knocked in the bacterial lacZ reporter under the control of the Spegα and Apeg-1 promoters. In these mice, Speg isoforms were expressed predominantly in cardiomyocytes during the developmental period (Liu et al., 2009). Just prior to birth there was evidence for lacZ staining in large elastic arteries, but there was no evidence of expression in the coronary vessels. These data demonstrated that the primary defect leading to the cardiomyopathy in Speg^−/−^ newborn mice was in the cardiomyocytes. In the present study we further assessed adult mice. Immunostaining of adult hearts revealed expression of Speg in cardiac vessels (**Figure 9A**), however, staining for CD31 in Speg^+/−^ mice (in the presence or absence of TAC) showed no differences in the number of vessels per mm^2^ of cardiac tissue compared with Speg^+/+^ mice (**Figure 9B**). All of the vessels appeared patent and without stenosis histologically, arguing that the focal areas of replacement fibrosis were not due to a lack of blood supply, but rather an intrinsic abnormality of Speg^+/−^ cardiomyocytes when exposed to the increased demand of pressure overload. Further evidence for a primary cardiomyocyte abnormality is the fact that Speg-KO mice, with targeted disruption of the Spegα and Spegβ in striated muscle, demonstrated a comparable dilated cardiomyopathy in adult mice (**Figure 2**). Additional findings in the Speg^+/−^ mice after TAC are reminiscent of decompensated heart failure due to pressure overload with evidence of cardiomyocyte cell death (Dorn, 2009), and collagen accumulation with interstitial fibrosis (Spinale, 2007; Kehat and Molkentin, 2010).

Recently we demonstrated that a significant defect in Speg^−/−^ hearts in neonatal pups is a lack of cardiomyocyte maturity. In comparison with Speg^+/+^ hearts, cardiomyocytes from Speg mutant mice showed a lack of striations and a less organized appearance (Liu et al., 2015). The myofibrils were much thinner and loosely arranged, and the cytoplasm of the Speg^−/−^ cardiomyocytes appeared less dense. This was confirmed by electron microscopy, with the volume fraction of myofibrils greatly reduced in Speg^−/−^ myocytes (Liu et al., 2015), supporting the notion that Speg is required for myocyte maturation.

While Speg^−/−^ mice have a dramatic cardiac phenotype consisting of a dilated cardiomyopathy with perinatal lethality (Liu et al., 2015), the consequences of a reduced, but not absent, expression of Speg was not known. Speg^+/−^ mice did not have cardiac abnormalities under basal conditions. However, haploinsufficiency of Speg leads to an impaired compensatory response after prolonged pressure overload of 4 weeks, resulting in a dilated cardiomyopathy. These findings are similar to titin (TTN), another member of the MLCK family. TTN is a very large sarcomeric protein that links the Z-disk with the M-line, and like the Unc-89 family, contains many immunoglobulin and fibronectin type 3 (FN3) domains (Temmerman et al., 2013). The generation of a TTN knock-in mouse, with a 2 base pair insertion to promote a premature stop codon, resulted in defects in sarcomere formation and death in early embryonic life (embryonic day 9.5) (Gramlich et al., 2009). Similar to Speg^+/−^ mice, TTN haploinsufficient mice did not develop a cardiac phenotype under basal conditions. Interestingly, pressure overload by TAC also resulted in a significant decrease in LV function, and an increase in cardiac fibrosis compared with wild-type mice after TAC. Our present findings, along with prior reports, suggest that deficient expression of MLCK family members may lead to fetal/newborn death, and that even reduced expression in the setting of a “second hit” – such as pressure overload – is adequate to induced decompensated heart failure.

Taken together, our data support the concept that beyond the perinatal period, the expression of Speg is vital for adult cardiac homeostasis, and also for the response to pathophysiologic stress, such as pressure overload. The importance of Speg has also been proposed in human hearts, as expression of Speg is decreased in human end-stage heart failure (Quick et al., 2017). However, at the present time, it is unknown whether reduced expression of Speg in humans contributes to, or is a consequence of, cardiac dysfunction.

## Author Contributions

CS, HH, YX, AS, YP, VH, and XL performed the experiments. CS, HH, YX, MR, RP, PA, XL, and MP analyzed the data and interpreted the results of experiments. XL and MP conception and design of experiments. CS, XL, and MP manuscript writing and figure preparation. XL and MP approved final version of manuscript.

## Conflict of Interest Statement

The authors declare that the research was conducted in the absence of any commercial or financial relationships that could be construed as a potential conflict of interest.
