# Supplementary material for: Pressure Overload in Mice With Haploinsufficiency of Striated Preferentially Expressed Gene Leads to Decompensated Heart Failure
Source: Front Physiol. 2018 Jul 10;9:863. doi: 10.3389/fphys.2018.00863 (PMC6048438; doi:10.3389/fphys.2018.00863)
Supplement: Supplementary file 1 [file Data_Sheet_1.PDF]

Transverse Aortic Constriction

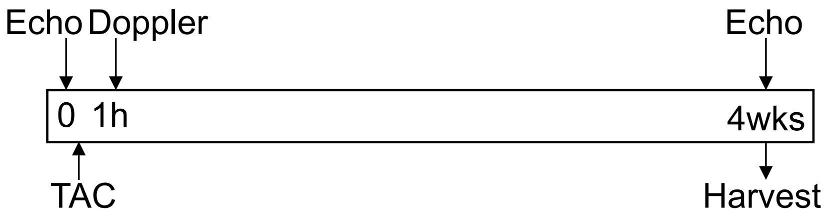

	Ahead Banding mmHg	Behing Banding mmHg	Gradient mmHg
	126	66	60
	145	75	70
	134	72	62
**Speg$^{+/+}$**	138	85	53
	**135.8**	**74.5**	**61.3**
	130	75	55
	145	70	75
	135	80	55
**Speg$^{+/-}$**	135	82	53
	146	76	70
	**138.2**	**76.6**	**61.6**

Supplementary Figure 1. Schematic of experimental protocol for transverse aortic constriction (TAC), and pressure gradient across the constriction. The upper panel shows a schema for the TAC, and subsequent echocardiograms. A baseline cardiac echocardiogram was performed in Speg$^{+/-}$ and Speg$^{+/+}$ mice, followed by TAC. Doppler analysis was acutely performed to confirm blood flow across the constriction. The next day, in a subgroup of animals, catheters were placed in the right and left carotid arteries, to assess the pressure gradient across the area of TAC (lower table). In the remainder of mice, either Speg$^{+/-}$ or Speg$^{+/+}$, echocardiograms were performed at 4 weeks after TAC (upper panel).
