# Supplementary material for: Pressure Overload in Mice With Haploinsufficiency of Striated Preferentially Expressed Gene Leads to Decompensated Heart Failure
Source: Front Physiol. 2018 Jul 10;9:863. doi: 10.3389/fphys.2018.00863 (PMC6048438; doi:10.3389/fphys.2018.00863)
Supplement: Supplementary file 2 [file Data_Sheet_2.PDF]

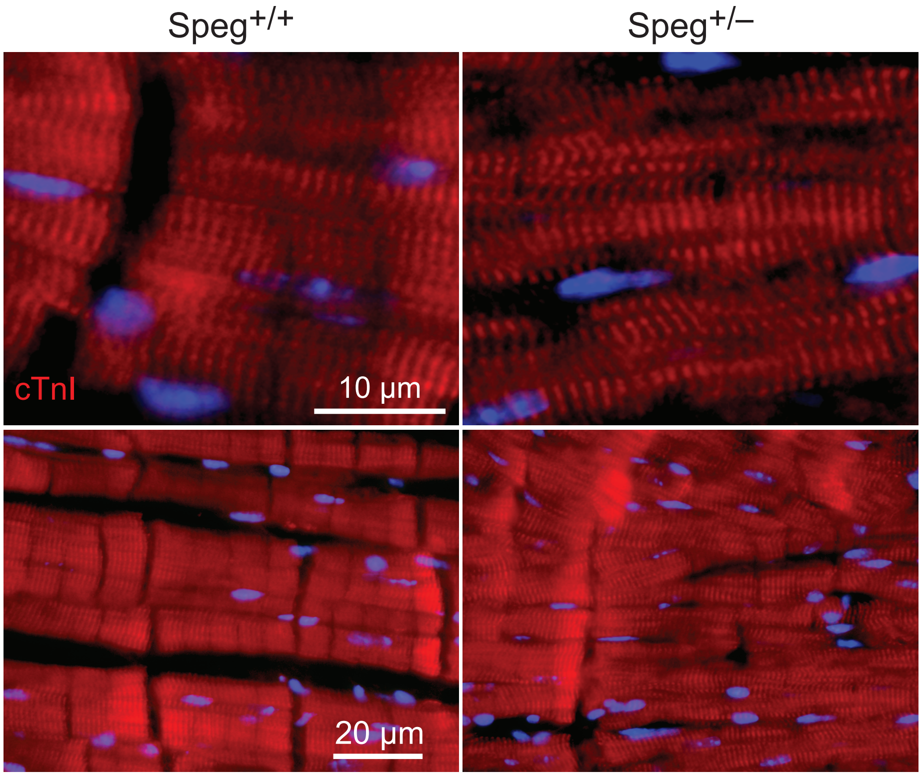

Supplementary Figure 2. Structure of Speg+/+ and Speg+/– cardiomyocytes in adult mouse hearts. Representative images of fluorescent microscopy for cardiac troponin I (cTnI, red) staining of cardiomyocytes from the left ventricle of Speg+/+ and Speg–/– hearts of adult mice. The hearts were co-stained with 4',6-diamidino-2-phenylindole (DAPI, blue) to identify nuclei. Scale bars, 10 µm (upper panels) and 20 µm (lower panels).
